# Detection of *BRCA1/2* mutations in circulating tumor DNA from patients with ovarian cancer

**DOI:** 10.18632/oncotarget.20722

**Published:** 2017-09-08

**Authors:** Magdalena Ratajska, Magdalena Koczkowska, Monika Żuk, Adam Gorczyński, Alina Kuźniacka, Maciej Stukan, Wojciech Biernat, Janusz Limon, Bartosz Wasąg

**Affiliations:** ^1^ Department of Biology and Medical Genetics, Medical University of Gdansk, Gdansk, Poland; ^2^ Laboratory of Clinical Genetics, University Clinical Centre, Gdansk, Poland; ^3^ Department of Pathology, Medical University of Gdansk, Gdansk, Poland; ^4^ Department of Gynecologic Oncology, Gdynia Oncology Center, Gdynia, Poland

**Keywords:** ovarian cancer, ctDNA, BRCA1/2, PARP1 inhibitor, next-generation sequencing

## Abstract

Approximately 25% of patients with ovarian cancer harbor a pathogenic *BRCA1/2* mutation that has been associated with favorable responses for targeted therapy with poly (ADP-ribose) polymerase 1 (PARP1) inhibitors compared to wild-type individuals. The overall frequency of germline and somatic *BRCA1/2* alterations is estimated at 13-15% and 3-10%, respectively. A high incidence of *BRCA1/2* somatic variants significantly increases the number of patients eligible for treatment with PARP1 inhibitors. Here, we assessed circulating tumor DNA (ctDNA) from 121 patients with ovarian cancer for *BRCA1/2* mutational analysis by next generation sequencing. A total number of patients carrying the pathogenic *BRCA1/2* variants was 30/121 (24.8%), including 22 and 7 individuals with exclusively germline or somatic mutations, respectively and one patient with variants of both origin. Among this cohort, more than one known pathogenic *BRCA1* and/or *BRCA2* alterations were identified in 7/30 individuals. The most recurrent mutations were detected in the *BRCA1* gene: c.5266dupC (p.Gln1756Profs^*^74) with the frequency of ~18%, followed by c.3756_3759del (p.Ser1253Argfs^*^10) and c.181T>G (p.Cys61Gly). In seven (5.8%) patients, coincidence of two or more *BRCA1/2* pathogenic mutations have been identified. Our results clearly demonstrate that the detection of both germline and somatic *BRCA1/2* mutations in ctDNA from ovarian cancer patients is feasible and may be a valuable complementary tool for identification of somatic alterations when the standard diagnostic procedures are insufficient. Finally, ctDNA can potentially allow to monitor the efficacy of PARP1 inhibitors and to detect a secondary reversion *BRCA1/2* mutations.

## INTRODUCTION

Ovarian cancer is one of the most common neoplasms among the European women, with more than 60,000 new cases diagnosed annually [1]. Approximately 13-15% of both unselected and familial ovarian cancer appear to be due to germline *BRCA1/2* mutations [2, 3], while somatic alterations were found in the additional 3-10% of cases [4–8]. In total, about one-fifth of ovarian cancer patients carry a pathogenic variant in the *BRCA1/2* gene. This worldwide frequency is comparable to the overall prevalence of *BRCA1/2* mutations among ovarian cancer patients in Polish population, estimating at ~15% for germline [9] and ~4% for somatic [10] variants.

A promising therapy with poly (ADP-ribose) polymerase 1 (PARP1) inhibitors has recently been widely studied in ovarian cancer patients [11, 12]. It has been demonstrated in clinical trials that *BRCA1/2* mutation carriers are eligible for this targeted therapy by having a better response compared with wild-type individuals [13–15]. Therefore, identification of somatic mutations in the *BRCA1/2* gene could expand the number of patients that may eventually benefit from treatment with PARP1 inhibitors.

The emergence of next-generation sequencing (NGS) has revolutionized the approach to diagnostic procedures in personalized medicine. Recently, this method has been successfully implemented as a highly sensitive and cost-effective diagnostic tool to detect either germline or somatic mutations, even in degraded DNA such as from formalin fixed paraffin embedded (FFPE) material. However, due to the wide heterogeneity of tumor cells, low quality of the extracted DNA and its potential contamination with DNA from non-neoplastic cells, the analysis of FFPE tumor material can be challenging. Thus, there is a clear clinical need to develop rapid, cost-effective and non-invasive tools for mutation screening in cancer and consequently implement them as standard diagnostic procedures.

Circulating tumor DNA (ctDNA), initially reported in 1948 by Mandel and Metais [16], is defined as a fraction of fragmented DNA derived directly from the tumor and circulated in the patient's blood. Briefly, ctDNA that is obtained in so-called “liquid biopsy” provides the representative information of all subclones in a tumor, including the presence of gene alterations. Recently, numerous studies have investigated the clinical value of liquid biopsy as potential diagnostic material. However, almost none of the research referred to the detection of *BRCA1/2* mutations in the plasma from ovarian cancer patients. To date, only Christie et al. (2017) reported the potential clinical utility of ctDNA analysis in 30 individuals with high-grade serous ovarian cancer [17]. However, this study aimed on the identification of reversion *BRCA1/2* mutations that could be responsible for acquiring the chemotherapy resistance, not on the *overall* screening of both germline and somatic alterations in a large cohort of unselected ovarian cancer patients.

Here, for the first time we established the frequency of the germline and somatic *BRCA1/2* mutations in 121 ctDNA samples from unselected ovarian cancer patients by using the comprehensive mutational analysis with NGS. Our results clearly indicate the potential clinical utility of ctDNA in the diagnosis of the ovarian cancer patients. Proposed approach allows to identify simultaneously germline and somatic variants and results in the increased number of patients who are potentially eligible for PARP1 inhibitors treatment. In addition, we discussed the technical aspects of ctDNA analysis that hamper its clinical implementation in personalized oncology.

## RESULTS

**Figure 1:**
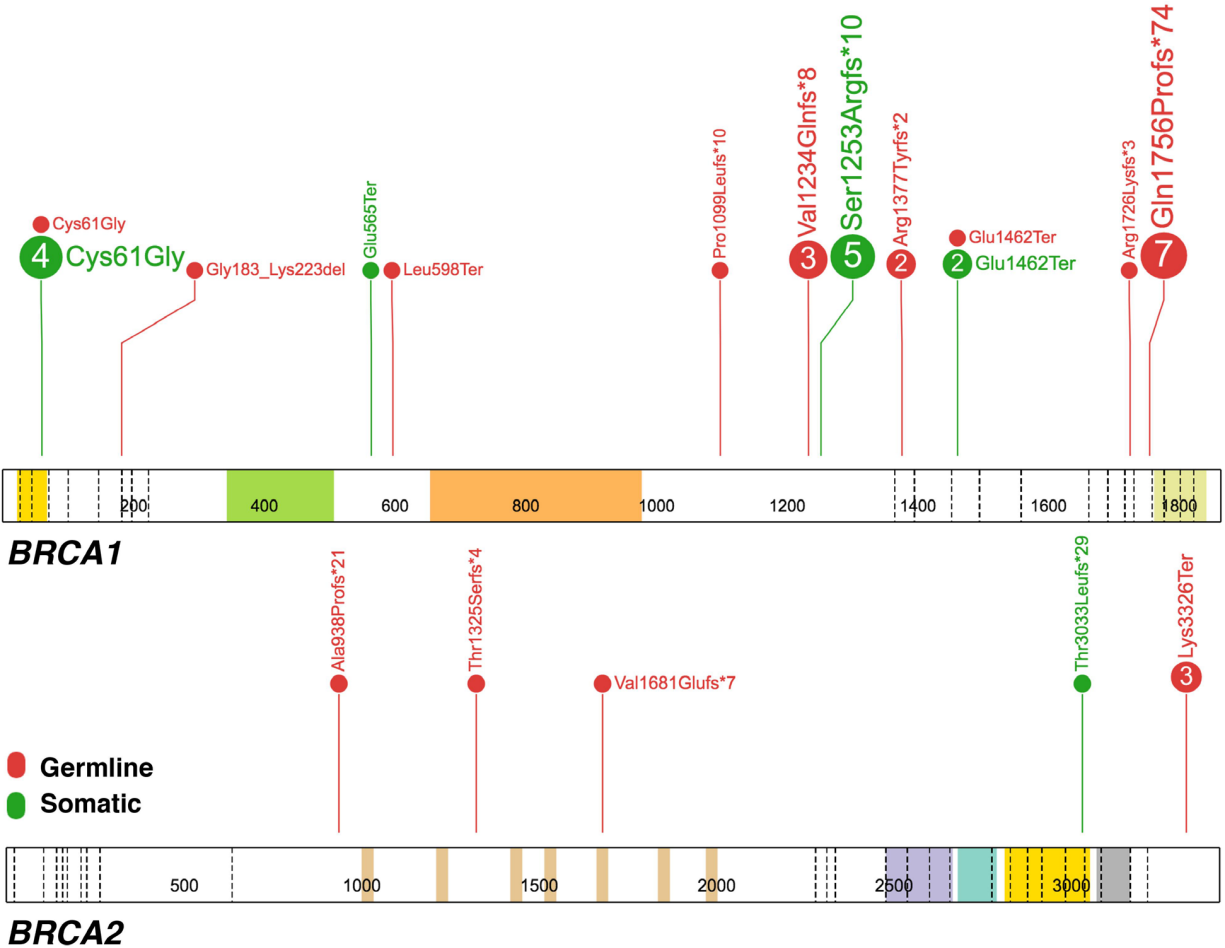
The spectrum of pathogenic germline and somatic mutations in the *BRCA1* and *BRCA2* genes detected in 121 ctDNA samples from ovarian cancer patients Each number in the circle corresponds with the total number of samples with specific alteration. The germline or somatic origin of mutation is depicted with the red and green color code, respectively. The figure was prepared using the ProteinPaint application [31].

Initially, 134 patients were enrolled to the study. ctDNA extraction was successful for 131 (98%) samples, while mutational analysis could be performed for 121 (90%) individuals. In this cohort, the pathogenic germline and somatic variants were identified in 23 (19%) and 8 (6.6%) patients, respectively. In the current study, we confirmed presence of all germline mutations that were identified in our previous study [9]. A total number of the positive *BRCA1/2* variants was 38, including 25 germline and 13 somatic alterations. In seven (5.8%) patients, coincidence of more than one pathogenic *BRCA1/2* mutations has been observed. Two individuals (#78 and #92) were classified as a double *BRCA1* and/or *BRCA2* mutations carrier, one patient (#8) had both germline and somatic *BRCA1/2* variants and in the remaining four patients we reported two (#169, #170, #171) or three (#175) different somatic *BRCA1* alterations. In total, the *BRCA1/2* mutations, either germline and/or somatic were detected in 30/121 (24.8%) individuals, including one patient carrying variants of both origin. The most recurrent alteration was c.5266dupC (p.Gln1756Profs^*^74) in the *BRCA1* gene, detected in 18.4% (7/38). Interestingly, in four individuals (3.3%) somatic p.Cys61Gly substitution, well-known Polish founder mutation, was detected. To confirm the presence of somatic p.Cys61Gly variant, DNA from FFPE tissues was extracted in duplicates and mutational analysis was performed using PCR followed by HRM in selected samples (#171 and #175). Mutation was detected in both samples; however, in sample #171 p.Cys61Gly variant was identified only in one tumor section, while the second was negative. All pathogenic *BRCA1/2* mutations reported in the current study are shown in Table 1 and Figure 1.

**Table 1:** Germline and somatic pathogenic *BRCA1/2* mutations identified in ctDNA from 121 patients with ovarian cancer

Gene	Mutation in corresponding ctDNA	Predicted effect	Type	Case no	FIGO stage	Origin	RS number	Classification ^A^	% reads
***BRCA1***	c.181T>G	p.Cys61Gly	M	#71	IIIC	Somatic	rs28897672	Pathogenic [class 5]	7
				#115	IIIC	Germline			51
	c.1793T>A	p.Leu598Ter	N	#85	IA	Germline	rs80357118	Pathogenic [class 5]	51
	c.3296delC	p.Pro1099Leufs^*^10	FS	#397	IIIC	Germline	rs80357815	Pathogenic [class 5]	38
	c.3700_3704del	p.Val1234Glnfs^*^8	FS	#22	IIIB	Germline	rs80357609	Pathogenic [class 5]	54
				#38	IIIC				50
				#66	IIIC				70
	c.3756_3759del	p.Ser1253Argfs^*^10	FS	#100	IIIC	Somatic	rs80357868	Pathogenic [class 5]	17
	c.5177_5180del	p.Arg1726Lysfs^*^3	FS	#162	IIIC	Germline	rs80357975	Pathogenic [class 5]	60
	c.5266dupC	p.Gln1756Profs^*^74	FS	#50	IIIC	Germline	rs397507247	Pathogenic [class 5]	43
				#108	NR				69
				#314	IIIC				49
				#323	IIIC				44
				#368	IV				46
				#374	IIIC				52
				#378	IIIB				52
	c.4357+2T>G	r.[=,4186_4357del] p.Arg1377Tyrfs^*^2	S	#95	IIIC	Germline	rs80358152	Pathogenic [NS]	49
	c.4484+1G>A	r.[=,4358_4484del] p.Glu1462Ter	S	#395	IIIC	Germline	rs80358063	Pathogenic [NS]	49
***BRCA2***	c.2806_2809del	p.Ala938Profs^*^21	FS	#164	IIIC	Germline	rs80359351	Pathogenic [NR]	50
	c.5042_5043del	p.Val1681Glufs^*^7	FS	#178	IIIC	Germline	rs80359478	Pathogenic [class 5]	49
	c.9097delA	p.Thr3033Leufs^*^29	FS	#168	IIIC	Somatic	rs762120301	Pathogenic [class 5]	16.5
	c.9976A>T	p.Lys3326Ter	N	#398	IV	Germline	rs11571833	? ^B^ [class 2]	44
				#411	IIIC				48
***BRCA1 (2 variants)***	c.[594-2A>C; 641A>G]	r.[=,594_670del] p.Gly183_Lys223del	S	#78	NR	Germline	rs80358033rs55680408	Low riskvariant [NS]	5252
	c.3756_3759del	p.Ser1253Argfs^*^10	FS	#169	IIIC	Somatic	rs80357868	Pathogenic [class 5]	10
	c.4484+1G>A	r.[=,4358_4484del]p.Glu1462Ter	S				rs80358063	Pathogenic [NS]	12
	c.1693G>T	p.Glu565Ter	N	#170	IIIC	Somatic	rs886039963	Pathogenic [class 5]	14
	c.3756_3759del	p.Ser1253Argfs^*^10	FS				rs80357868	Pathogenic [class 5]	20
	c.181T>G	p.Cys61Gly	M	#171	IIIC	Somatic	rs28897672	Pathogenic [class 5]	10
	c.3756_3759del	p.Ser1253Argfs^*^10	FS				rs80357868	Pathogenic [class 5]	10
***BRCA1 (3 variants)***	c.181T>G	p.Cys61Gly	M	#175	IIIC	Somatic	rs28897672	Pathogenic [class 5]	7
	c.3756_3759del	p.Ser1253Argfs^*^10	FS				rs80357868	Pathogenic [class 5]	4
	c.4484+1G>A	r.[=,4358_4484del] p.Glu1462Ter	S				rs80358063	Pathogenic [NS]	5
***BRCA1 & BRCA2 (2 variants)***	c.181T>G	p.Cys61Gly	M	#8	IV	Somatic	rs28897672	Pathogenic [class 5]	21
	c.3974_3975insTGCT	p.Thr1325Serfs^*^4	FS			Germline	-	Absent ^C^ [NR]	47
	c.4357+2T>G	r.[=,4186_4357del] p.Arg1377Tyrfs^*^2	S	#92	IIIC	Germline	rs80358152	Pathogenic [NS]	48
	c.9976A>T	p.Lys3326Ter	N			Germline	rs11571833	? ^B^ [class 2]	50

^A^ Variants’ classification reported in the publicly available databases, including the BIC database, BRCA Share^™^ database, ClinVar and/or the HGMD; variants’ classification reported in the BRCA Exchange database curated by the ENIGMA consortium was presented in square brackets; ^B^ this variant should be re-classified in the publicly available databases from prior benign/little clinical significance to likely pathogenic based on the recent findings [18]; ^C^ reported as novel pathogenic variant in 1/144 female patient with hereditary breast cancer, but no co-segregation study provided [30]; however, this variant fulfilled the followings ACMG recommendations [29]: PVS1, PM2, PM4, PP3 and PP4, thus it can be clearly classified as pathogenic. FS: frameshift; N: nonsense; M: missense; S: splice; NR: not reported; NS: presence of the variant in the database, but its clinical significance has not yet been specified.

All detected *BRCA1/2* variants have been previously reported as pathogenic in the publicly available disease databases (BIC database, BRCA Share™ database, BRCA Exchange database, ClinVar and/or the Human Genome Mutation Database, last accessed May 2017), except one *BRCA2* variant (c.9976A>T; Lys3326Ter) that was initially submitted as benign with little clinical significance. However, in line with the recent findings [18], this alteration should be re-classified from the prior benign to likely pathogenic interpretation.

## DISCUSSION

To assess the usefulness of ctDNA in screening for *BRCA1/2* mutations, we performed next-generation sequencing in a group of 121 unselected patients diagnosed with ovarian cancer. The overall frequency of germline and somatic *BRCA1/2* mutations found in the plasma from ovarian cancer patients was 19% and 6.6%, respectively that is consistent with the previous studies [2–7, 19, 20]. As expected, all germline *BRCA1/2* mutations, identified previously in constitutional DNA [9], were detected in circulating DNA, thereby showing 100% specificity and sensitivity of the ctDNA analysis for germline mutations. A slightly higher prevalence of germline alterations (19% vs. 13-15%) was observed in the studied cohort compared to worldwide estimates; however, that is still comparable with our recent reports [9–10]. Indeed, the population's ethnic background with a specific founder mutation and/or the application of various molecular approach with the distinct detection rates might explain these differences in frequency. As for somatic variants, 13 known pathogenic mutations were identified in the additional ~7% of patients that is higher than in our previous report (4.1%) [10]. This difference in frequency of somatic mutations among individuals with the same ethnic background might be explained by intratumoral heterogeneity. In contrast to FFPE sample, which represents only a subpopulation of the whole tumor, analysis of ctDNA provides a comprehensive view of tumor, including all subclones in the primary tumor as well as in distant metastases. That was demonstrated in this study by identifying more than one somatic pathogenic *BRCA1/2* variants in four ctDNA samples (Table 1). The presence of intratumoral heterogeneity was also observed while confirming the p.Cys61Gly somatic variant in DNA extracted from two different sections of #171 tumor tissue; the mutation was detected only in one section. Therefore, we strongly believe that ctDNA can give new insights into pathogenesis of ovarian cancer and response of the patients to PARP1 inhibitors.

Among the positive *BRCA1/2* variants, c.5266dupC (p.Gln1756Profs^*^74) and c.181T>G (p.Cys61Gly), both located in the *BRCA1* gene, were the most common mutations detected in 18.4% and 13.2% of the studied group, respectively (Figure 1). Both alterations are well-known founder mutations in the Polish population [20, 21]. Interestingly, in the current cohort the c.181T>G was predominantly reported as somatic mutation (Table 1). To date, somatic p.Cys61Gly mutation was detected only in one patient [4]. Presence of germline and somatic variants at the same region may suggest the existence of potential mutational hotspot location. Laitman et al. (2013) reported that the *BRCA1* c.68_69delAG (p.Glu23Valfs), one of the most common founder mutations among Ashkenazi women, is not only limited to this population; this alteration arises independently more than once in non-Jewish cohorts, at least in Malaysia and the UK populations [22]. These results support the mutational hotspot theory and may explain the existence of alike variants, both germline and somatic origin.

In addition, we identified the presence of two germline *BRCA1* variants c.594-2A>C and c.641A>G (p.Asp214Gly), located in *cis* position, in a single patient (#78). For a long time, it has been suggested that splice variant c.594-2A>C should be considered as a high-risk pathogenic mutation, because it causes exon 10 skipping. Recently, de la Hoya et al. (2016) demonstrated that *BRCA1* c.[594-2A>C;641A>G] is predicted to be a low-risk pathogenic alteration [23].

Despite the promising potential of ctDNA analysis for mutation screening of cancer-related genes, this approach has several limitations. The major drawback of this procedure is the high degree of ctDNA fragmentation and its low concentration in the circulation. A total amount of ctDNA can greatly vary from 0.01% to even 90% depending on the histological type and cancer clinical stage [24]. Although it has been widely accepted that serum contains higher concentrations of ctDNA (at least 2-4 times) than plasma, plasma is the preferably material source, especially in the field of oncology [25]. This increase in serum is likely due to the contamination with constitutional DNA from blood cells during the clotting process. Another technical aspect, the optimal handling protocols, is necessary to avoid hemolysis that might result in high rate of the false negative results. The use of dedicated cell-free DNA blood collection tubes, the proper transport and storage conditions and the optimal time between blood collection and plasma separation will unequivocally minimalize the risk of hemolysis [26, 27]. In addition, the knowledge about the origin and biological mechanisms of ctDNA release is limited. For instance, it is still unclear why the size of plasma DNA samples differs between the several cancer types. Generally, ctDNA is highly fragmented with a median size of 170 bp, but larger fragments (~332 bp and/or ~498 bp) can also be observed in a subset of cancer patients [27, 28]. Taken together, these technical aspects critical for ctDNA analysis, related mostly to pre-analytical procedures, should be validated before its implementation in routine practice.

In this study, we demonstrated that detection of both germline and somatic *BRCA1/2* mutations in circulating DNA is feasible and might be helpful as a complementary tool for identification of somatic alterations when the standard diagnostic procedures with using FFPE samples are insufficient. Most tumors are characterized by genomic heterogeneity and due to its clonal evolution, they prone to acquire resistance regardless of the type of therapy. In this context, ctDNA analysis might be used to repeatedly monitor tumor's genomic profile, providing an early warning of its recurrence long before it will be clinically noted. Taken together, ctDNA analysis might be a powerful opportunity for the diagnosis, prognosis and management of cancer patients; however, several pivotal pre-analytical and analytical aspects need to be standardized before its clinical implementation.

## MATERIALS AND METHODS

### Patients and sample collection

Among the previously studied group of consecutive 134 patients with ovarian cancer referred for *BRCA1/2* genetic testing [9], ctDNA was extracted successfully from the plasma of 131 individuals. All samples for molecular testing were collected prior to therapy. The histological diagnosis of ovarian cancer was evaluated by pathologists, including 72% of individuals with stage III or IV serous ovarian cancer (Table 1). The average patient age at diagnosis was 59 years (range: 27-87). Blood samples that were collected in Cell-Free DNA BCT ® tubes (Streck) were processed within 24 hours after collection and were centrifuged to separate the plasma from the peripheral blood cells. Due to the high risk of contamination with germline material, samples with hemolysis (n=10) were excluded from further analysis. In total, 121 ctDNA samples with an average ctDNA input of 5.5 ng (range: 0.5-39.2 ng) were sequenced.

The research was approved by the local ethics committee at the Medical University of Gdansk. All patients provided informed written consent prior to study enrollment.

### DNA extraction

Circulating tumor DNA was extracted from the separated plasma using cobas cfDNA Sample Preparation Kit (Roche Molecular Diagnostics) according to the manufacturer's protocol. DNA from FFPE tumor tissues with cellularity more than 70% was isolated with cobas DNA Sample Preparation Kit (Roche Molecular Diagnostics). Quantity and quality of isolated ctDNA was determined with Qubit Fluoremeter (ThermoFisher Scientific) and 2100 Bioanalyzer (Agilent Technologies), respectively.

### Mutational analysis

*BRCA1* and *BRCA2* mutation screening in ctDNA samples was performed using the *BRCA Tumor MASTR Plus* assay (Multiplicom) according to the manufacturer's protocol. MiSeq targeted resequencing ~1000 to 2000x (Illumina) was performed and 4% cut-off for the Variant Allele Frequency was applied. The analysis was performed with Sophia DDM (Sophia Genetics), Geneious (Biomatters Ltd.) and Alamut (Interactive Biosoftware) softwares.

The presence of pathogenic somatic *BRCA1/2* mutations in ctDNA was confirmed by the independent NGS analysis of second samples. All patients had done the comprehensive analysis of the *BRCA1/2* gene testing in the peripheral blood samples as part of our previous study [9], thereby the additional confirmation of germline variants detected in ctDNA was not necessary. The status of somatic variants in FFPE tissue samples was ascertained by high resolution melting (HRM) analysis (Idaho Technology Inc.).

The nomenclature of the alterations was based on *BRCA1* mRNA sequence NM_007294.3 and *BRCA2* mRNA sequence NM_000059.3 according to the recommendations of the Human Genome Variety Society (http://varnomen.hgvs.org/). Variants’ interpretation pathogenicity was performed in line with the American College of Medical Genetics (ACMG) recommendations [29].

## Abbreviations

ctDNA: circulating tumor DNA
FFPE: formalin fixed paraffin embedded
NGS: next generation sequencing
PARP1: poly (ADP-ribose) polymerase 1
